# Contextual determinants of generational continuation of female genital mutilation among women of reproductive age in nigeria: analysis of the 2018 demographic and health survey

**DOI:** 10.1186/s12978-024-01778-1

**Published:** 2024-03-26

**Authors:** Tosin Olajide Oni, David Aduragbemi Okunlola

**Affiliations:** 1 Department of Demography and Social Statistics, Obafemi Awolowo University, Ile-Ife, Nigeria; 2 Department of Sociology, College of Social Sciences and Public Policy, Florida State University, Tallahassee, Florida USA

**Keywords:** Female genital mutilation, Generational continuation, Nigeria, Childbearing women, Community

## Abstract

**Background:**

Female genital mutilation (FGM) has negative health implications and has long been recognised as violating sexual rights. Despite the huge efforts expended on eradicating FGM, generational continuation of the practice, i.e. the act of mutilated women also mutilating their daughters, persists in Nigeria. This study investigated the individual, household, and community factors associated with generational continuation of FGM among women in Nigeria.

**Methods:**

The study analysed data from the 2018 Nigeria Demographic and Health Survey (NDHS). A weighted sample of 3835 women with FGM history and who had given birth to female children was analysed. Models were estimated using mixed-effects multilevel logistic regression with Stata 16.0.

**Results:**

The results showed that 40.0% of women continued FGM for their daughters. Regional prevalence of FGM continuation ranged from 14.9% in the South-South (the lowest) to 64.3% in the North-West (the highest). Women aged 15–24 years (uaOR = 0.40; 95% CI:0.28–0.57) and rich (uaOR = 0.44; 95% CI:0.35–0.56) had the least likelihood of generational continuation of FGM. In communities with low proportions of women unexposed to the media, the likelihood of FGM continuation was significantly higher (uaOR = 1.85; 95% CI:1.35–2.53). Generational continuation of FGM was significantly lower in communities with moderate proportions of uneducated mothers (aOR = 0.6; 95% CI:0.42–0.86).

**Conclusion:**

FGM continuation was high in Nigeria, and it was most common among older and poor mothers and in communities with large proportions of uneducated women and those unexposed to the media. Existing National Policy and Plan on FGM elimination should be strengthened to target these characteristics.

**Supplementary Information:**

The online version contains supplementary material available at 10.1186/s12978-024-01778-1.

## Background

Female genital mutilation (FGM) refers to a partial or complete removal of a female's external genitalia and other injuries to the genitals for non-medical reasons [1]. The origin of FGM is traceable to Egypt where it was majorly practised on enslaved people to make sex difficult or impossible in order to safeguard them from unwanted pregnancies [2]. There are four major types of FGM [3]: Type I involves the partial or total removal of the clitoris, and this is called clitoridectomy; Type II is called excision and it involves the cutting of either of or both labia majora and labia minora, in addition to what is involved in Type I (removal of the clitoris); Type III is called infibulation and it entails narrowing down the vaginal orifice and creating a covering seal on it; whilst Type IV is more generic, involving any harmful practice such as piercing, pricking or scraping of the female genitalia. FGM is an inhumane and dangerous procedure that violates the principles of gender equality and non-discrimination [3, 4].

FGM undermines the right of women to physical integrity because sensitive sex organs are cut off/mutilated [5]. It violates respect because it is mainly done at a time when girls are unable to give informed consent [6]. Also, it is a threat to wellbeing and health because it leads to extended bleeding and complicated infections, which might result in death [7]. FGM is usually performed on girls/women in groups using the same instrument throughout the process (in an unsterile environment) especially when carried out by religious and cultural leaders [8, 9]. This leads to numerous medical, economic and social implications (7, 10]. For instance, WHO reports that no less than $4 million is spent annually on treating obstetric complications associated with FGM [10]. Despite the numerous issues associated with FGM and several regional and sub-regional interventions, laws and declarations that have described FGM as an infringement on human rights [1, 3], the practice persists [11].

In Africa and the Middle Eastern countries, no less than 200 million girls and women have undergone FGM by 2017 [11]. Nearly 33 million of the mutilated girls and women in these regions are from Nigeria and this represents about 13% of the country’s female population aged below 40 [11]. The prevalence of FGM in Nigeria ranks far higher than the rate in neighbouring countries of Ghana and Uganda, both of which have FGM rate of 1% [11]. Recognising the negative impacts of FGM, the Nigerian government formulated a National Policy and Plan of Action for the Elimination of FGM (NPPFGM) in 2013 [12]. The policy targeted the reduction of FGM from 30% in 2008 to 5% by 2017 through several national responses and programs. Although the programmes made significant progress in raising awareness and reporting the negative consequences of FGM [10], data from the 2018 Nigeria Demographic and Health Survey (NDHS) show that the rate of FGM is at 19.2% [13]. This indicates that the target was far from being met. In 2021, another NPPFGM (2021–2025) was developed to consolidate on the initial Plan and to achieve the targeted maximum FGM prevalence of 5%. For this policy to deliver on target, there is a need for further research to identify the underlying drivers of FGM.

A previous study shows that mothers' intentions and perceived benefits of FGM underlie their intention to practice genital mutilation [14]. This may relate to mothers who ascribe sexual purity to FGM and for them, performing FGM on their daughters could help them to maintain the 'legacy' of sexual purity [15, 16]. Where mothers who were themselves mutilated in turn expose their daughters to the same mutilation process, this is referred to as generational continuation of FGM in this study. The Social Convention Theory, developed by Mackie [17], posits that it is conventional for mothers who underwent FGM and saw nothing bad in it to extend it to their daughters. Beyond this theoretical postulation, it might be reasoned that some mothers who underwent FGM might find it harmful and thus refuse to extend it to their daughters. However, available evidence from Nigeria shows that there are factors that might make women to expose their daughters to FGM despite the harms posed by the practice [14–16]. For instance, many girls/women are made to undergo FGM out of fear of stigmatisation and rejection in their communities [16]. In some parts of Nigeria, FGM is essential to girls' marriageability and religious inclination [8]. However, while many Christian, Muslim and traditional religious leaders have condemned FGM [18, 19], others have expressed support for it [20, 21]. Moreover, a handful of healthcare workers support the practice of FGM [22], despite their supposed knowledge of the associated health complications.

Based on the foregoing, there are probable influences of some firmly held pro-FGM values, perceptions and attitudes which contextually exist in households and communities in Nigeria. Studies have reported how perceptions and some contextual factors such as poverty and low education prevailing in communities serve as drivers of FGM practice (24–26). Available evidence also shows that in households where female inferiority is reinforced through patriarchy, the likelihood of subjecting women/girls to FGM is significantly higher [23]. However, many of these previous studies focused on women of reproductive age in general, many of whom might not even be aware that they were mutilated. Despite the plethora of studies investigating women’s FGM practice for their daughters [23–25], there is paucity of research examining FGM practice for daughters among women who themselves have undergone FGM. Arguably, mutilated women are likely to be aware of the negative consequences of FGM such that the factors that would make them expose their daughters to such consequences are likely to be beyond personal, but that which extends to some household and community influences. However, this remains unknown in view of paucity of evidence. Against this backdrop, this study investigated the household and community-level factors associated with generational continuation of FGM.

## Methods

### Study design

The study utilized data from the 2018 Nigeria Demographic and Health Surveys (NDHS). The data was used with written permission from ICF, which is the implementer and primary owner of Demographic and Health Surveys (DHS) conducted in countries around the world. DHSs are funded by the United States Agency for International Development (USAID) in collaboration with individual countries. The surveys adopt a cross-sectional design with multi-stage, clustered, and stratified sampling designs, which rely on the most recent census of the individual countries as the sampling frame. The 2018 NDHS adopted a multi-stage sampling with the use of Enumeration Areas designed for the 2006 National Population Census by the Nigeria Population Commission (NPC). The sampling design used the 36 States and the Federal Capital Territory as strata while the local government areas (LGAs) within each State were used as clusters. Eligible households and individuals were selected within clusters stratified into rural and urban, thus making the survey nationally representative. The NDHS dataset has many recode files which include the individual recode (referring to women aged 15–49 years), household, men and kids’ recodes. Further details on the methodologies used for the implementation of DHSs are available on dhsprogram.com.

### Data source

The Individual Recode file from the 2018 NDHS were analysed in this study. The individual recode contains information of 41,821 women of reproductive age. Information contained in the recode includes family planning, maternal and child health and nutrition, childhood mortality, malaria, female genital cutting, sexual activity, marriage, and sexually transmitted infections NPC & ICF Macro, 2019).

### Target population

In this study, 3,835 women of reproductive age (15–49 years) who had female children and reported that they (the mothers) had undergone FGM were extracted from the survey dataset. These inclusion criteria were necessary to examine generational continuation of FGM. The group of women who met the inclusion criteria and who provided responses to questions on FGM were created as a ‘sub-pop’ and used for analysis from the dataset.

### Measures

- Outcome variable

In this study, the outcome variable was the generational continuation of FGM. The variable was measured in the NDHS by asking eligible women about the "number of daughters circumcised". Women who reported '0' (no daughter circumcised) were classified as "not continued", while others who reported at least one (> 0) were classified as (1) "continued". Those who provided no response to this question were not included in the sup-pop analysed for this study.

- Explanatory variables

The main explanatory variables in this study were household and community-level socio-demographic and economic variables. Others were individual and household-level variables. Their selection was guided by previous studies [26–28]. The community-level variables used in this study were the type of place of residence (urban and rural) and region of residence (North-Central, North-East, North-West, South-East, South-South and South-West). Other variables were computed by aggregating the women's individual or household-level variables at cluster levels. The resulting scores across the clusters were subsequently categorised into tertiles (low (25%), middle (50%) and high (75%). Community poverty was computed using cluster-level aggregation of the poorest and poorer categories of women, and the scores were trichotomised as low, moderate, and high. A similar approach was used to derive the proportions of uneducated women, uneducated male partners, women unexposed to media, women decision-makers in their households and support for FGM in communities.

The proportion of women belonging to each of the major ethnic groups in Nigeria (Yoruba, Hausa, Igbo) was aggregated at the cluster level and dichotomised as low (1) and high (2). The same approach was used to compute the proportions of Igbo and Hausas/Fulani women in the communities. Cluster-level aggregation of variables (and subsequent dichotomising or trichotomizing) has been used in previous studies [26, 27].

The household-level variables were wealth index, female household headship (yes [headed by a woman] or no [headed by a man] and women’s household decision-making ability. The wealth index—computed by the DHS programme based on respondents' assets in their households – as quintiles groups (20% each): poorest, poorer, middle, richer and richest—was re-categorised as poor, middle, and rich. Women's decision-making ability was captured in the NDHS using five questions that asked about who made decisions on (a) how to spend respondent’s income and earnings, (b) respondent’s health and health-seeking, (c) large purchases in the household, (d) visits to family or relatives and (e) what to do with money husband earns. Those who responded with "husband/partner alone" to the questions were categorised into 'low' (i.e. they had low decision-making ability), those who chose 'respondent alone' were grouped into 'high' (they had high decision-making ability) while others (joint decisions) were grouped as 'medium'.

The individual level variables were women’s age (15–24, 25–34, and 35–49 years), years of education, exposure to mass media, religion (Christianity, Islam, and others), ethnicity (Hausa/Fulani, Igbo, Yoruba and others). The women’s age categorization was based on their pregnancy risk profile, in which pregnancies among women aged 35 + are regarded as high risk while those of women aged 15–24 are regarded as early pregnancy [29]. Support for FGM at the individual level was measured by asking women if "FGM should be continued or stopped" and the response options were *continued*, *stopped*, *depends* and *don't know*. Those who chose "stopped" were grouped as those who did not support FGM (0), while others were deemed to support FGM’s continuation (code as 1).

### Data analysis

All variables were described using frequencies, percentages or and medians (interquartile ranges). The hierarchical structure of the NDHS, the binary nature of generational continuation of FGM and this study's interest in the contextual correlates of generational continuation of FGM justified fitting multilevel logistic regression. This model has been widely used in examining the hierarchical correlates of demographic, social and population health phenomena because the effects of predictor variables could vary between communities, thus violating the assumption of independence of residuals in linear regression [27, 30, 31]. Five regression models were fitted: the first model (null model, not shown) contains only the intercept term while models 2 to 4 are unadjusted models which examined the bivariate relationships between each of the individual, household and community level variables and generational continuation of FGM. The fifth model (adjusted) shows the multivariate influences of the individual, household and community factors on the outcome variable.

The multilevel logistic regression is expressed as:

1 $${\mathbf{Y}}_{{\text{ij}}}={{\varvec{\upbeta}}}_{00}+{{\varvec{\upbeta}}}_{{\text{ij}}}{\mathbf{X}}_{{\text{ij}}}+{\mathbf{U}}_{0{\text{j}}}+{\mathbf{e}}_{{\text{ij}}}$$

where:

${{\text{Y}}}_{{\text{ij}}}$ = log-odds of generational continuation of FGM.

${\upbeta }_{00}$= Fixed intercept (average of Yij).

${{\varvec{\upbeta}}}_{\mathbf{i}\mathbf{j}}$ = Coefficients for individual, household and community level variables for a woman "i" in "j" community.

${{\text{X}}}_{{\text{ij}}}$ = Individual, household and community level variables for a woman "i" in "j" community.

$${\mathbf{U}}_{0\mathbf{j}}$$ = Random intercept.

${{\text{e}}}_{{\text{ij}}}$ = Residuals for the individual, household and community-level variables for a woman "i" in "j" community.

Data analysis was performed using Stata 16.0 [32]. Given the complex sampling design adopted in the implementation of the NDHS, the "*subpop*" command from the Stata software was used to restrict analyses to women aged 15–49 who had daughters and had undergone FGM. This was done so that the statistical technique would be able to compute valid standard errors from the survey data [33]. Adjusted Odds Ratios (aOR), representing the marginal effect of the explanatory variables on generational continuation of FGM (and their 95% confidence intervals), were produced from the regressions. The significance of the aORs was tested against a 5% level of significance. Interclass Correlation Coefficient (ICC), ranging from 0 to 1 (the higher, the better), was also computed (as the random effect component of the regression model) to show the proportion of variation in generational continuation of FGM due to between-cluster differences. Log-likelihood tests of the regressions were tested to show their goodness of fit.

## Results

Table 1 shows that 40% of women continued FGM. The women’s average number of years of education was six. Women aged 35–49 constituted the highest proportion (47.2%), while the lowest (18.3%) were aged 15–24. Concerning religion, 57% and 42% of women practised Islam and Christianity, respectively. The ethnic composition was such that 40.26% of women were Hausa/Fulani, 26% were Yoruba, and 20% were Igbos. At the household level, 44% of women were rich, while 36% were poor. Results also show that very few (8.8%) households were female-headed. Residential composition shows that 52% of women were urban residents. The highest proportion of women was from the North-West (35.7%), and the least was from the North-East region (5.5%). Based on cluster-level aggregations, the results show that about 37% of the communities had a high proportion of uneducated mothers while 39.2% had a low proportion of women unexposed to media.

**Table 1:** Description of study variables

Variables	*N* = 3835	%	Median
**Generational continuation of FGM**			
Continued	1535	40.0	
Not continued	2300	60.0	
**Individual level**			
**Women's characteristics**			
Years of education			6 years
Exposure to mass media			2 times (weekly)
**Age**			
15–24	702	18.3	
25–34	1321	34.5	
35–49	1812	47.2	
**Religion**			
Christianity	1627	42.4	
Islam	2198	57.3	
Others	10	0.2	
**Ethnicity**			
Hausa/ Fulani	1544	40.3	
Igbo	776	20.2	
Yoruba	996	26.0	
Others	519	13.5	
**Male partner's characteristics**			
Age			43 (years)
Years of education			6 (years)
**Household-level factors**			
**Wealth Index**			
Poor	1378	35.9	
Middle	753	19.6	
Rich	1704	44.4	
**Female household headship**			
Yes	339	8.8	
No	3496	91.2	
**Female decision-making ability in HH**			3
**Community-level factors**			
**Type of place of residence**			
Urban	2001	52.2	
Rural	1834	47.8	
**Region**			
North Central	291	7.6	
North East	209	5.5	
North West	1371	35.7	
South East	668	17.4	
South South	351	9.2	
South West	945	24.7	
**Proportion of Yorubas**			
Low	2441	63.7	
High	1394	36.4	
**Proportion of Igbos**			
Low	2398	62.5	
High	1437	37.5	
**Proportion of Hausas**			
Low	1912	49.9	
High	1923	50.2	
**Proportion of uneducated mothers**			
Low	1351	35.2	
Medium	1054	27.5	
High	1430	37.3	
**Proportion of uneducated male partners**			
Low	1217	31.7	
Medium	1157	30.2	
High	1461	38.1	
**Community poverty**			
Low	1420	37.0	
Medium	1226	32.0	
High	1889	31.0	
**Proportion unexposed to media**			
Low	1504	39.2	
Medium	1306	34.1	
High	1025	26.7	
**Proportion of women decision-makers in household**			
Low	615	16.0	
Medium	1683	43.9	
High	1537	40.1	
**Community support for FGM**			
Low	2370	61.8	
Medium	1230	32.1	
High	234	6.1	

*IQR* *interquartile range*

Results in Table 2 show that women aged 15–24 were 60% less likely to continue FGM than their counterparts aged 35–49 years (uaOR = 0.40; CI: 0.277 – 0.569) [Model 2]. Less of Christian respondents (22.7%) than Muslim respondents (53.0%) continued FGM and it was also shown that Christians were 39% less likely than Muslims to continue FGM (uaOR = 0.61; CI: 0.452 – 0.837). Among the ethnic groups, the Hausas had the highest (63.7%) proportion of women who continued FGM, compared with the Igbos (25.4%) and the Yorubas (23.5%). Hausas were about 3 times more likely to continue FGM than women from other ethnic groups (uaOR = 3.84; CI: 2.762 – 5.340). Similarly, 35.1% of women who supported FGM continued it and they had an 8% higher likelihood of generational continuation of FGM. As shown in Model 3, women from middle-income level and rich households were 32.3% (uaOR = 0.67; CI: 0.511 – 0.877) and 56% (uaOR = 0.44; CI: 0.348 – 0.556) less likely to continue FGM. This indicates that the higher the household wealth, the lower the likelihood of continuing FGM in such households. In addition, results showed that female involvement in household decisions had a significant influence on the generational continuation of FGM. In households with female involvement in decision-making, there was 14% less likelihood of FGM continuation (uaOR = 0.86; CI: 0.814 – 0.911).

**Table 2:** Bivariate analysis of individual, household and community effects on generational continuation of FGM

**Individual factors**		% with continued FGM	Individual Factors (*model 2*)	Household Factors (*model 3*)	Community Factors (*model 4*)
			uaOR (95% C.I)	uaOR (95% C.I)	uaOR (95% C.I)
Age	15–24	42.8	0.40 (0.28–0.57)^a^		
	25–34	44.9	1.05 (0.83–1.33)		
	35–49	35.4	0^b^		
Years of Education			0.99 (0.96–1.02)		
Religion	Christianity	22.7	0.61 (0.45–0.84)^a^		
	Islam	53.0	0^b^		
Ethnicity	Yoruba	23.5	0^b^		
	Hausa	63.7	3.84 (2.76–5.34)^a^		
	Igbo	25.4	1.04 (0.75–1.45)		
	Others	23.1	0.90 (0.64–1.28)		
Personal support for FGM	Yes	35.1	1.08 (1.03- 1.13)^a^		
	No	16.7	0^b^		
Level of exposure to Media			0.99 (0.98–1.00)		
**Household factors**					
Wealth status	Poor	54.2		0^b^	
	Middle	43.4		0.67 (0.51–0.88)^a^	
	Rich	27.1		0.44 (0.35–0.56)^a^	
Female household headship	Yes	31.2		0.93 (0.69–1.25)	
	No	40.9		0^b^	
Female involvement in household decision	Yes	39.4		0.86 (0.81–0.91)^a^	
	No	43.3		0^b^	
**Community factors**					
Zone	North Central	37.7			1.42 (0.91–2.23)
	North East	59.8			3.54 (1.72–7.26)^a^
	North West	64.3			3.57 (1.80–7.06)^a^
	South East	25.9			1.27 (0.73–2.23)
	South South	14.9			0.67 (0.40–1.11)
	South West	20.5			0^b^
Residence	Urban	29.8			0.84 (0.65–1.08)
	Rural	51.2			0^b^
Proportion of Yoruba	High	26.5			0^b^
	Low	47.7			1.59 (1.02–2.48)^a^
Proportion of Igbo	High	24.1			1.10 (0.79–1.53)
	Low	49.6			0^b^
Proportion of Hausa	High	63.3			1.82 (1.03–3.21)^a^
	Low	22.3			0^b^
Proportion of the poor in the communities	Low	25.2			0^b^
	Moderate	42.2			1.41 (0.92–2.16)
	High	55.5			1.38 (1.01–1.88)^a^
Proportion of uneducated mothers	Low	23.8			1.18 (0.63–2.20)
	Moderate	29.3			0.63 (0.44–0.90)^a^
	High	63.3			0^b^
Proportion of uneducated partners	Low	23.6			0^b^
	Moderate	29.9			1.02 (0.72–1.43)
	High	61.7			1.35 (0.79–2.33)
Proportion unexposed to media	Low	20.9			0^b^
	Moderate	46.9			1.96 (1.43–2.70)^a^
	High	59.4			1.44 (0.91–2.26)
Proportion of women participants in household decision	Low	48.1			0^b^
	Moderate	45.7			1.11 (0.83–1.50)
	High	30.6			1.21 (0.88–1.66)
Proportion of community women who supported FGM	Low	42.2			0^b^
	Moderate	37.2			1.73 (1.57–1.92)^a^
	High	33.3			1.75 (0.50–1.82)

^a^significant at 5%; uaOR  unadjusted odds ratio, *CI*  *confidence interval; b. is set at zero for the reference category*

Generational continuation of FGM was most prevalent in the North West (64.3%), followed by the North East (59.8%) but least in the South-South (14.9%). Results on community factors showed that women in the North West and North East were both about 3 times more likely to continue FGM (uaOR = 3.54; CI: 1.724 – 7.257 and uaOR = 3.57; CI: 1.803 – 7.060 respectively than their counterparts in the South West zone. Women in communities with a low proportion of Yoruba had a 59% higher likelihood of continuing FGM than women in communities with a high proportion of Yoruba (uaOR = 1.59; CI: 1.020 – 2.484). Conversely, however, women in communities with a high proportion of Hausa had an 82% higher likelihood of continuing FGM than women in communities with a low proportion of Hausa (uaOR = 1.59; CI: 1.020 – 2.484). Furthermore, women in communities with a high proportion of poor were 38% more likely than women in communities with a low proportion of poor to continue FGM (uaOR = 1.38; CI: 1.008 – 1.875). Also, women in communities with a moderate proportion of individuals unexposed to the media were 96% more likely than women in communities with a low proportion of individuals unexposed to the media to continue FGM (uaOR = 1.96; CI: 1.427 – 2.701). Similarly, communities with moderate support for FGM were 73% more likely to experience continuation of FGM than communities with low support for FGM (uaOR = 1.73; CI: 1.571 – 1.923).

Table 3 presents results in the full model. The results show that age was a significant factor influencing generational continuation of FGM as women aged 15–24 years were 56% less likely than women of older age (35–49 years) to continue FGM (aOR = 0.34; CI: 0.239 – 0.486). Women who had support for FGM were also 9% more likely to continue FGM. In households where females were involved in decision-making, there was a 9% less likelihood of FGM continuation among women. Following model adjustment, the North East and North West regions became the zones with the highest likelihood (3.95 and 3.66) of FGM continuation (aOR = 3.66; CI: 1.717 – 7.812 and aOR = 3.95; CI: 1.937 – 8.039 respectfully). With a moderate proportion of uneducated mothers in a community, a continuation of FGM was 40% less likely than in communities with a high proportion of uneducated mothers. Also, with a moderate proportion of individuals unexposed to the media in a community, the continuation of FGM was 85% (aOR = 1.85; CI: 1.347 – 2.532) more likely than in communities with a low proportion of individuals unexposed to the media. Furthermore, in communities with low levels of support for FGM, there was 24% less likelihood of FGM continuation than in communities with high community support for FGM.

**Table 3:** Contextual determinants of generational continuation of FGM (Full Model)

**Individual factors**		aOR (95% C.I.)
Age (in years)	15–24	0.34 (0.24—0.49)^a^
	25–34	1.01 (0.80—1.27)
	35–49	0^b^
Years of Education		1.00 (0.97—1.02)
Religion	Christianity	0.89 (0.64—1.23)
	Islam	0^b^
Ethnicity	Yoruba	1.17 (0.62—2.23)
	Hausa	0.64 (0.32—1.25)
	Igbo	0.72 (0.44—1.17)
	Others	0^b^
Personal support for FGM	Yes	1.09** (**1.04—1.15)^a^
	No	0^b^
Level of Exposure to Media	Low	0.99 (0.98—1.00)
	Medium	0.99 (0.97—1.01)
	High	0^b^
**Household factors**		
Wealth Status	Poor	1.05 (0.76 1.46)
	Middle	0.88 (0.63—1.25)
	Rich	0^b^
Female Household Headship	Yes	1.15 (0.87—1.52)
	No	0^b^
Female Involvement in Household Decision	Yes	0.91 (0.85—0.96)^a^
	No	0^b^
**Community factors**		
Zone	North Central	1.55 (0.99—2.42)
	North East	3.66 (1.72—7.81)^a^
	North West	3.95 (1.94—8.04)^a^
	South East	1.79 (0.89—3.60)
	South South	0.88 (0.50—1.53)
	South West	0^b^
Residence	Urban	0.81 (0.63—1.03)
	Rural	0^b^
Proportion of Yoruba	High	1.41 (0.89—2.24)
	Low	0^b^
Proportion of Igbo	High	1.17 (0.83—1.66)
	Low	0^b^
Proportion of Hausa	High	1.38 (0.71—2.67)
	Low	0^b^
Proportion of the Poor in the communities	Low	0.93 (0.67—1.31)
	Moderate	0.67 (0.41—1.09)
	High	0^b^
Proportion of Uneducated Mothers	Low	1.14 (0.59—2.20)
	Moderate	0.60 (0.42—0.86)^a^
	High	0^b^
Proportion of Uneducated Partners	Low	1.03 (0.74—1.44)
	Moderate	1.16 (0.66—2.04)
	High	0^b^
Proportion Unexposed to Media	Low	1.85 (1.35—2.53)^a^
	Moderate	1.47 (0.93—2.33)
	High	0^b^
Proportion of Women Participants in Household Decision	Low	1.13 (0.84—1.52)
	Moderate	1.35 (0.97—1.87)
	High	0^b^
Proportion of Community Women who supported FGM	Low	0.76 (0.51—1.14)
	Moderate	0.70 (0.55—0.90)^a^
	High	0^b^

^a^significant at 5%; aOR adjusted odds ratio, *CI* *confidence interval; b. is set at zero for the reference category*

## Discussion

The study examined the prevalence and associated contextual factors of generational continuation of FGM in Nigeria. The study found a 40% FGM continuation level, which is much higher than the overall level reported (19.5%) in the 2018 NDHS [13] but similar to the levels reported in other studies that also examined women’s FGM practice for daughters [14, 15]. The disparity in the reported level of FGM in this study and that of other studies that also used NDHS data is likely due to the difference in focus. While the current study focused on mutilated women who have daughters, those other studies either focused on women in general [30] or only on women who have daughters [14]. Focusing on mutilated women who had female birth history allowed the study to identify how women's personal experience with FGM influenced their decision to extend it to their daughters, given specific contextual situations. This gave impetus to the study's adoption of the social convention theory, which was used to evaluate the rate of continuation of FGM. Another previous study also used the theory to provide information on how social norms influenced the practice of FGM in migration contexts [34].

Irrespective of the focus or the method used, however, this study confirms that FGM practice remains high in Nigeria as women continue to expose their daughters to the practice. For women who had experienced FGM to extend it to their daughters, a plausible explanation is that the FGM they had might not be humiliating, especially if done by trained medical personnel. In some conservative Nigerian communities, it is not an uncommon situation for mothers to tell their daughters how FGM helped them remain virgins before meeting their husbands (the daughter's father) [35]. Where mothers hold such beliefs, they are likely to expose their girls to the same FGM treatment. While it may not be palatable for mothers to willingly expose their daughters to the same painful FGM experience they had, they may do so if they believe that the 'gains' would outweigh the 'pains'. After all, studies have shown women report benefits of FGM as 'promoting' marital fidelity and premarital fertility [35, 36], despite its known-no-health-benefit status [1].

The study further reveals that some individual and household level factors were significant correlates of women's FGM continuation. At the individual level, young women (aged 15–24) had 56% significantly less likelihood while those who supported FGM were 9% more likely to continue FGM. Young women may refuse to expose their daughters to FGM for obvious reasons, some of which have also been reported in earlier studies [30, 37]. One, the younger generation are often in a stage wherein they explore romantic relationships and sexual pleasures [38]. In the course of this exploration, their memory of the pains and limited sexual pleasure that typically characterise FGM [37] is likely to remain fresh. This is unlike for older women who may have rationalised and/or outgrown such unpalatable memories. Two, younger women are likely to be closer to their fellow younger and yet-to-be-married counterparts who may be in the habit of sharing information on sexual pleasures, all of which may be different from their own experience, given their FGM history. As a result, such young women may not only question their parents on why they did FGM for them, but may also disconnect themselves from its continuation.

At the household level, this study shows that wealth status had a significant influence on FGM continuation. These results are in consonance with many previous studies in which women with high economic status reportedly had a lower FGM prevalence [39, 40]. A significantly lower FGM rate among daughters of rich women is plausibly due to the ability of such women to negotiate what they want for their daughters with husbands/in-laws/extended family, who may be pushing for FGM. Conversely, the result is in contrast with another study that reported a significantly positive relationship between economic status and FGM levels among women [41]. Whatever the case, however, that women who had the resources (wealth) to protect their daughters from FGM would prefer to expose them to it is a pointer to many things. One, their support for FGM might be deep-rooted in personal beliefs and pro-FGM cultures, as also reported in another study [42]. This assumption is compelling because the women themselves were mutilated and they could have prevented their daughters from a similar experience, if not for personal beliefs in its 'benefits'. This result also lends credence to the social convention theory [17, 34]. Two, given the women's wealth, which would enable them to afford health facility care, it was likely that the FGM performed on their daughters was carried out by skilled professionals. To them, this limits the health risks associated with FGM, thus justifying their FGM continuation decision. This is supported by a study which shows economic status as a salient motivator for the medicalisation of FGM [41, 42].

The study shows that female household headship had no significant influence in reducing the rate of FGM among daughters. A plausible reason for this is that women who are household heads in a patriarchal setting like Nigeria's might apply extreme measures to ensure that the daughters raised in the households are not 'social deviants' or promiscuous. A part of that extreme caution may be to expose their daughters to FGM so that premarital sex becomes intolerable, thereby making premarital pregnancy less likely. While daughters in male-headed households are likely to be blamed for their own misbehaviour, patriarchal society may be quick to blame the female head for the misbehaviour of daughters in female-headed households. Studies have shown that family structure and headship have important influences on specific outcomes such as homicide rate, delinquency and expected social behaviours [43, 44]. However, there is no sufficient evidence to conclude that female household heads throw off their daughters' sexual health for the sake of social desirability.

At the community level, results show that FGM continuation was significantly less in communities with a higher proportion of young and educated mothers. In other words, women's education became a significant factor in dissuading women from continuing FGM in communities where they were in high proportion. This result is consistent with previous studies that reported the significant role of education in eliminating FGM [45, 46]. In communities where a high proportion of women were involved in household decision-making, there was no significant commitment to discontinue FGM. Again, this confirms the bivariate level results which show that women did not leverage their decision-making ability to prevent their daughters from FGM. However, with a moderate-to-high proportion of educated women in communities, education became a tool to prevent daughters from FGM. The significant role of maternal education in protecting children from common unhealthy community practices such as FGM, infant delivery at home and infant weaning before 6 months are well established in the literature [27, 46].

The study also shows that having a low proportion of women exposed to the media in communities significantly increases the likelihood of FGM continuation. This is consistent with previous studies that reported the influence of media exposure on FGM practice [47, 48]. This is because several interventions, including the National Policy and Plan of Action for the Elimination of FGM that are committed to eradicating FGM have leveraged on using the media to change the narratives of pro-FGM beliefs, opinions and perceptions [12, 49]. Where women are exposed to such anti-FGM media campaigns, they may save their daughters from FGM, even if they themselves were mutilated. However, given that the media contents to which women were exposed are not specified in the NDHS data, no conclusion could be drawn in this study on the effect of media exposure on generational continuation of FGM.

The study utilised nationally representative data that is publicly accessible (via dhsprogram.com). Other researchers can validate the study's results by examining the study variables and associated documentation. Standard procedures were used during the NDHS data collection process and this was deemed as an assurance of quality. The study’s use of cluster-level aggregation to generate community-level variables enabled the comparison of the outcome variable across levels of community-level factors. However, the retrospective nature of the NDHS data may lead to inaccurate inferences because the retrospective reporting might be inaccurate due to recall errors. Also, it might not be ruled out that daughters who have not been mutilated as at the time of the NDHS data collection would still not be exposed to FGM later in life. This is because the age at FGM exposure differs and ranges widely from childhood to beyond adolescence in Nigeria [50]. Thus, the prevalence of generational continuation of FGM in this study might have been underreported. Nevertheless, the findings are an accurate representation of the phenomenon up to the period that the analysed data were collected. Lastly, while we could establish the temporality of the explanatory variables, the cross-sectional nature of the NDHS data would not permit any cause-and-effect relationship claim. This explains our preferential use of 'influence' rather than 'effect' in the multivariable analysis.

The study filled an important knowledge gap by providing information on the household and contextual factors associated with generational continuation of FGM in Nigeria. The findings provide more evidence to support the formulation of relevant policies and the implementation of the existing ones. One takeaway from the study is that for the National Policy and Plan of Action for the Elimination of FGM (2021–2025) to deliver on target, the cooperative involvement of all tiers of government and non-governmental organisations is required to tackle the household and community enablers of FGM. Interventions aimed at eradicating FGM in Nigeria should be targeted at older women because they are the major drivers of the practice. The interventions will be more effective if specific attention is paid to women and girls in female-headed households. The study also provides evidence to support interventions aimed at women’s education and community sensitisation against exposing girls to FGM. Irrespective of the experience of mothers who had FGM, it is important to inform them that it is harmful to continue same with their daughters. Given the time-limitation of the cross-sectional design used in the NDHS, longitudinal investigations are required to follow-up mutilated women in order to examine their level of generational continuation of FGM up to when their daughters reach adulthood.

## Code availability

The Stata script (dofile) used for the analysis is available upon request. The corresponding author should be contacted for this.

## Conclusion

Generational continuation of FGM is high in Nigeria and is mainly reinforced by older women and in communities with high proportion of poor women and those with low education. Interventions aimed at eliminating or reducing daughters’ exposure to FGM would be most effective where older mothers, poor and uneducated women are specifically targeted.

### Supplementary Information

**Supplementary Material 1.**

## Data Availability

The data analysed in this study are publicly available at https://www.dhsprogram.com

## Abbreviations

FGM: Female Genital Mutilation
GC: Generational Continuation
NPPFGM: National Policy and Plan of Action for the Elimination of FGM
NDHS: Nigeria Demographic and Health Survey
