# Supplementary material for: Contextual determinants of generational continuation of female genital mutilation among women of reproductive age in nigeria: analysis of the 2018 demographic and health survey
Source: Reprod Health. 2024 Mar 26;21:39. doi: 10.1186/s12978-024-01778-1 (PMC10964639; doi:10.1186/s12978-024-01778-1)
Supplement: Supplementary file 1 — Supplementary Material 1. [file 12978_2024_1778_MOESM1_ESM.docx]

Model goodness of fits

| Models | Description | Log-likelihood | p^†^ | ICC |
| --- | --- | --- | --- | --- |
| Model 1 | Null | -2.274 | <0.0001 | 0.805 |
| Model 2 | Individual-level only | -2.106 | <0.0001 | 0.32 |
| Model 3 | Household-level only | -2.232 | <0.0001 | 0.617 |
| Model 4 | Community-level only | -2.129 | <0.0001 | 0.688 |
| Model 5 | Full model | -2.005 | <0.0001 | 0.675 |

*^†^Based on the Log-likelihood ratio test; ICC: Intraclass Correlation Coefficient*
